# Supplementary material for: Bioenergetic and Cytokine Profiling May Help to Rescue More DCD Livers for Transplantation
Source: Int J Mol Sci. 2023 May 31;24(11):9536. doi: 10.3390/ijms24119536 (PMC10253900; doi:10.3390/ijms24119536)
Supplement: Supplementary file 1 [file ijms-24-09536-s001.zip › ijms-2402042-supplementary.pdf]

Table S1: SUIT01 protocol

Step	Compound	Concentration	Coupling control state	ET-pathway state	Function
1	Tissue homogenate (2 mL)	1 mg wet mass·mL-1		ROX	
2	ADP-Mg2+ (Merck, 117105)	5 mM		ROX	Substrate of ATP synthase
3	Malate (Sigma, M1000)	0.1 mM			NADH-linked substrate
4	Octanoylcarnitine (APExBIO, B6371)	0.5 mM	OXPHOS (P)	FAO	Substrate for fatty acid oxidation (FAO)
5	Cytochrome c (Sigma, C7752)	10 µM	OXPHOS (Pc)	FAO	Evaluate outer mt-membrane integrity
6	Pyruvate (Sigma, P2256)	5 mM	OXPHOS (Pc)	FAO+NADH	Feeds TCA (via Acetyl-CoA)
7	Malate (Sigma, M1000)	2 mM	OXPHOS (Pc)	FAO+NADH	NADH-linked substrate
8	Glutamate (Sigma, G1626)	10 mM	OXPHOS (Pc)	FAO+NADH	NADH-linked substrate
9	Succinate (Sigma, S2378)	10 mM	OXPHOS (Pc)	FAO+NADH+S	Substrate of Complex II
10	Rotenone (Sigma, R8875)	0.5 µM	OXPHOS (Pc)	S	Inhibition of Complex I
11	Antimycin A (Sigma, A8674)	2.5 µM		ROX	Inhibition of Complex III

ADP, adenosine diphosphate; ATP, adenosine triphosphate; ROX, residual oxygen consumption; FAO, fatty acid oxidation; NADH, nicotinamide adenine dinucleotide; S, succinate; TCA, tricarboxylic acid cycle

Table S2: SUIT02 protocol

Step	Chemical	Concentration	Coupling control state	Function
1	Tissue homogenate (2 mL)	1 mg wet mass·mL^{-1}		
2	Rotenone (Sigma, R8875)	0.5 µM		Inhibition of Complex I
3	Succinate (Sigma, S2378)	10 mM	LEAK (*L*)	Substrate of Complex II
4	ADP Mg^{2+} (Merck, 117105)	5 mM	OXPHOS (*P*)	Substrate of ATP synthase
5	Cytochrome *c* (Sigma, C7752)	10 µM	OXPHOS (P_c)	Evaluate outer mt-membrane integrity
6	CCCP (Sigma, C2759)	0.5 µM per step	ET (*E*)	Uncoupling of mitochondrial respiration
7	Antimycin A (Sigma, A8674)	2.5 µM		Inhibition of Complex III

ADP, adenosine diphosphate; ATP, adenosine triphosphate; CCCP, carbonyl cyanide m-chlorophenyl
